# Effect of Rice (*Oryza sativa* L.) Ceramides Supplementation on Improving Skin Barrier Functions and Depigmentation: An Open-Label Prospective Study

**DOI:** 10.3390/nu14132737

**Published:** 2022-06-30

**Authors:** Teik Kee Leo, Eugenie Sin Sing Tan, Farahnaz Amini, Navedur Rehman, Edmond Siah Chye Ng, Chung Keat Tan

**Affiliations:** 1 Research and Development Department, Nexus Wise Sdn Bhd, Petaling Jaya 47400, Malaysia; tkleo@nexuswise.com; 2 School of Healthy Aging, Aesthetics and Regenerative Medicine, Faculty of Medicine and Health Science, UCSI University, Kuala Lumpur 56000, Malaysia; eugenietan@ucsiuniversity.edu.my (E.S.S.T.); farahnaz@ucsiuniversity.edu.my (F.A.); edmondng@ucsiuniversity.edu.my (E.S.C.N.); 3 Scunthorpe General Hospital, NHS, Yorkshire and Humber, Scunthorpe DN157BH, UK; navedur.rehman@nhs.net

**Keywords:** rice (*Oryza sativa* L.) ceramides, skin barrier function, transepidermal water loss (TEWL), wrinkle severity, pigmentation

## Abstract

Ceramides plays a crucial role in maintaining skin barrier function. Although foregoing evidence supported beneficial effects of topical ceramides for restoration of the skin barrier, studies on oral ceramides are extremely scarce, with most published data collected from in vivo and in vitro models. Thus, this study aimed to evaluate the efficacy of rice ceramides (RC) supplementation to improve skin barrier function and as a depigmenting agent through comprehensive clinical assessments. This study investigated the beneficial effects of orally administered RC supplementation in 50 voluntary participants. Skin hydration, firmness and elasticity, transepidermal water loss (TEWL), melanin index (MI), erythema index (EI), sebum production, pH, and wrinkle severity were assessed at baseline and during monthly follow-up visits. RC supplementation was found to significantly (*p* < 0.01) improve skin hydration, sebum production, firmness and elasticity, and wrinkle severity for three assessed areas, namely the left cheek, dorsal neck, and right inner forearm. Additionally, RC significantly (*p* < 0.01) reduced the rates of TEWL, levels of MI and EI. Analyses of data indicated that participants at older age were more responsive towards the effect of RC supplementation. Our findings suggest that RC supplementation can effectively improve skin barrier function, reduce wrinkle severity, and reduce pigmentation.

## 1. Introduction

Human epidermis is composed of five different layers. Stratum basale is the innermost layer, followed by stratum spinosum, granulosa, lucidum, and stratum corneum (the outmost layer). Stratum corneum, the primary mechanical barrier of the skin, is formed by multiple actions of lipid consisting of ceramides, cholesterol, and fatty acids in an approximately equal molar ratio of 1:1:1 [1]. All three components play an essential role in skin integrity; particularly, the ceramides are crucial for maintaining epidermal homeostasis [2]. Ceramides are chemically composed of long-chain sphingoid bases linked with free fatty acids via an amide bond, primarily synthesized in the stratum spinosum within the skin epidermis. The synthesized ceramides are eventually assembled into a lamellar structure filling up spaces between cells and creating a lipid-containing membrane that maintains competent skin barrier function. Also, ceramides promote epidermal self-renewal and regulate skin immune responses [3].

The content of skin ceramides declines with skin aging, reducing up to 30% of total skin lipid profile compared to young stratum corneum [4]. The alteration in skin lipid profiles leads to a defective permeability barrier, causing higher transepidermal water loss (TEWL). Thus, TEWL is regarded as an important indicator of skin integrity. Besides aging, skin ceramides are also influenced by sex, ethnicity, anatomical region, dietary intakes, season, smoking status, and others [5,6,7]. Atopic dermatitis (AD) is the most common chronic skin disease globally and is associated with skin barrier dysfunction due to the loss of rice ceramides [8]. Although the underlying mechanism has not yet been fully elucidated, a recent review has highlighted the potential involvement of Th1 and Th2 cytokines in suppressing ceramides synthesis [8]. Topical application of ceramides-based products have proven beneficial in treating AD as adjuvant therapy [9]. Besides AD, other skin conditions and diseases such as psoriasis, Netherton syndrome, ichthyoses, acne, pruritus, and even melanoma were found to be associated with ceramides abnormalities [10].

Generally, animal-sourced and synthetic ceramides have been widely used in many ceramides-based products due to their cost-effective and time-effective production [11]. Nonetheless, skin-similar ceramides isolated from edible botanical sources have recently gained much attention due to their safety compared to animal or synthetic origin [12]. Glucosylceramides, a glycoside of ceramides, are a major sphingo (glycol) lipid in plants. The beneficial effects of glucosylceramides for improving skin hydration and restoring skin barrier function have been established for plant sources such as konjac [13], beet [14], and wheat [15]. Although foregoing evidence supported beneficial effects of topical ceramides for restoration of the skin barrier, studies on oral ceramides are extremely scarce, with most the published data being derived from in vivo and in vitro models. Additionally, the potential benefit of ceramides as a depigmenting agent is mainly unexplored, with limited evidence from animal studies [16].

Therefore, this clinical study was undertaken to evaluate the efficacy of rice ceramides (RC) supplementation for improving skin barrier functions and as a depigmenting agent. The efficacy was evaluated via a comprehensive biophysical assessment of skin parameters including skin hydration, TEWL, skin firmness and elasticity, melanin index (MI), erythema index (EI), sebum production, and pH.

## 2. Materials and Methods

### 2.1. Study Design

**Figure 1:**
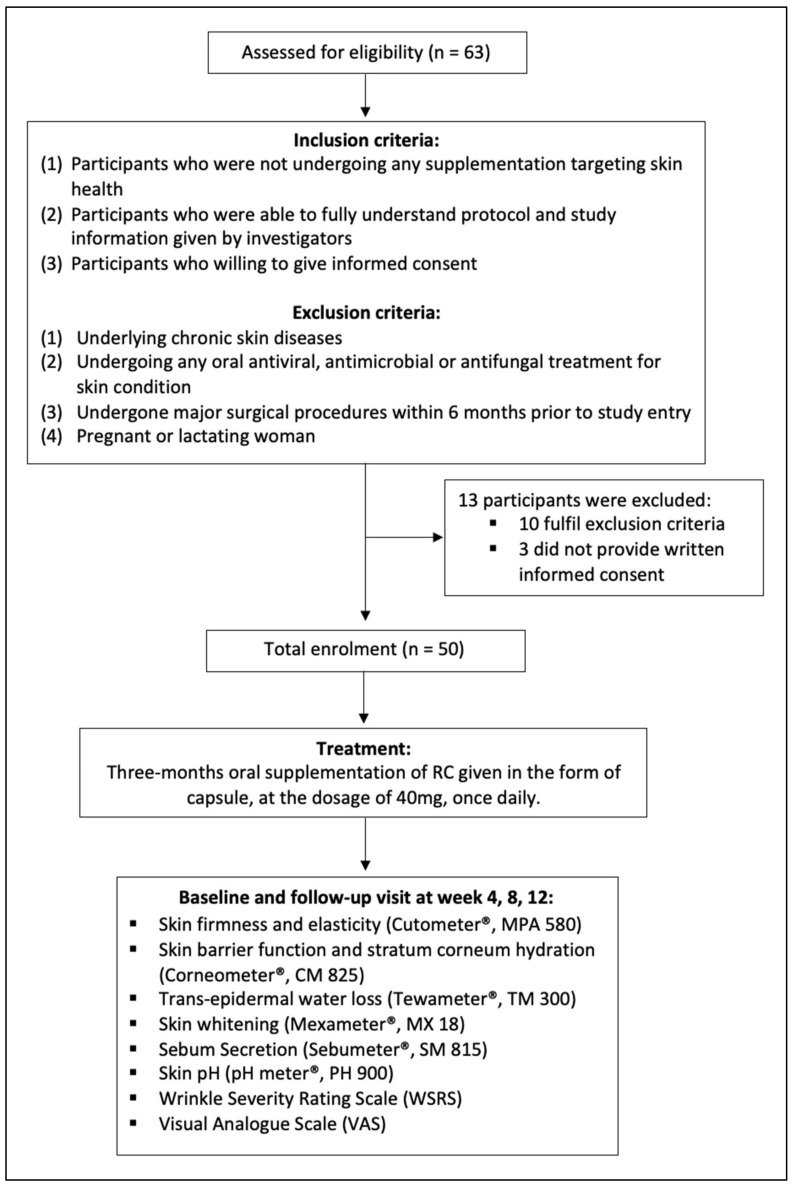
CONSORT protocol for the study described with flow diagram.

This is an open-label, single-arm, prospective study including three months of RC supplementation. The study was conducted in full compliance with the principles outlined in the Declaration of Helsinki and Malaysia’s Good Clinical Practice [17]. Eligibility of each participant was confirmed according to the protocol checklist, and their written informed consent was obtained. The study was approved by the principal investigator’s Institutional Ethics Committee (UCSI University, Malaysia, approval code IEC-2021-FMHS-022). The protocols of this study were registered in ClinicalTrials.gov with identifier NCT05101421. The CONSORT flow diagram is illustrated in Figure 1.

### 2.2. Participants Selection

Fifty volunteers (aged 21-year-old and above) with good general health conditions were recruited. Recruitment was conducted at UCSI University, Kuala Lumpur, Malaysia. All participants were provided with a participant information sheet and were given thorough explanations by the investigator. Written informed consent was sought from each participant. The following inclusion criteria were applied: (1) participants who were not undergoing any supplementation for skin health; (2) participants who had a good understanding of the study protocol and relevant study information provided by investigators; and (3) participants who granted their informed consent. Participants were excluded based on these exclusion criteria: (1) underlying chronic skin diseases; (2) undergoing any oral antiviral, antimicrobial, or antifungal treatment for a skin condition; (3) undergone major surgical procedures in the past six months prior to the study recruitment; and (4) pregnant or lactating women.

### 2.3. Supplementation

During the baseline visit, participants’ demographics and medical history were collected. Subsequently, participants began their three-month RC oral supplementation in the form of a capsule (KOMECERA^TM^, Nexus Wise, Selangor, Malaysia). The RC dosage per capsule was 40 mg and was to be taken once daily. Three monthly follow up visits were conducted during this study. A physical case report form (CRF) was utilized to record the participant’s self-evaluation for the severity of symptoms during each visit.

### 2.4. Skin Biophysical Measurements

Physiologic properties of skin were assessed using a non-invasive Multiple Probes Analyser (MPA) (Courage & Khazaka, Cologne, Germany). Skin firmness and elasticity were assessed using Cutometer (MPA 580; Courage & Khazaka, Cologne, Germany). The values were expressed in viscoelasticity unit (VEU), indicating resistance to the mechanical force versus ability to return. A value close to 1 indicates better elasticity and firmness. Transepidermal water loss (TEWL) was assessed using Tewameter (TM 300; Courage & Khazaka, Cologne, Germany) and rate of evaporation was recorded with its values expressed in g/h/m^2^. Skin barrier function and stratum corneum hydration was assessed with a capacitance based Corneometer (CM 825; Courage & Khazaka, Cologne, Germany), and values were expressed in arbitrary units (AU). Melanin index (MI) and erythema index (EI) were measured using Mexameter (MX 18; Courage & Khazaka, Cologne, Germany), and outcomes were expressed as index values for each parameter (MI and EI) in AU on a scale from 0 to 999. Sebum production was assessed based on grease spot photometry using Sebumeter (SM 815; Courage & Khazaka, Cologne, Germany), and values were also expressed in AU on a scale from 0 to 350. Surface pH was measured using a skin pH meter (PH 900; Courage & Khazaka, Cologne, Germany) equipped with a flat glass electrode (Mettler-Toledo, Giessen, Germany), and values were expressed in pH.

All measurements were conducted based on detailed methods and measuring principles outlined by Lim et al. [18]. Measurements were carried out on three precise skin areas: (1) left cheek, 2 cm below the orbit on the left side of the face in the inter-pupillary line; (2) right inner forearm, 3 cm above the elbow; and (3) dorsal neck, 3 cm below the spinous process of the neck. Measurements were performed in a controlled research facility with constant room temperature between 21 °C and 23 °C to prevent sweating and relative humidity between 40% and 45%. Before measurements, subjects acclimatized to the external environment for 30 min. Three consecutive readings were taken at each area, and their mean values were calculated. All measurements were taken between 12 pm to 5 pm in order to minimize variation due to circadian cycle.

### 2.5. Wrinkle Severity Rating Scale (WSRS)

The improvement in the appearance of facial wrinkle was assessed using Wrinkle Severity Rating Scale (WSRS). WSRS is a validated 5 point reference scale classifying nasolabial folds (NLFs) [19]. Two independent clinical aesthetic doctors (assessors) were provided with an identical set of images displaying frontal views of the lower face. After that, they rated the severity based on the 5-grade WSRS. Photographs of participants were obtained during baseline and three monthly follow-up visits. The NLFs were captured in a relaxed state rather than stretched in all the photographs. The standard definition of five WSRS grades is G1: no visible NLFs, G2: shallow but visible NLFs, G3: moderate deep NLFs, G4: very long and deep NLFs, and G5: extreme deep long NLFs. Assessors were provided with the standard definition of WSRS grades and additional visual reference materials. Each grading was enclosed with a set of three reference images. All assessments were blinded, and photographs of each participant were randomized to prevent assessor bias.

### 2.6. Visual Analogue Scale (VAS)

Participants were asked to self-evaluate their general skin condition during baseline and three monthly follow-up visits using the Visual Analogue Scale (VAS). VAS is a psychometric measuring instrument designed for patients to evaluate disease-related symptom severity subjectively. Thus, a rapid and statistically reproducible classification of symptoms severity was achieved. Previous studies have proven that VAS is a useful approach for assessing skin conditions and diseases [20]. During the assessment, participants were asked to rate their condition based on a 10-cm long horizontal VAS, of which 0 points indicated the least healthy skin condition and 10 points indicated the healthiest skin condition.

### 2.7. Statistical Analysis

Demographic characteristics were presented as categorical data, expressed in frequency and percentage. All outcomes were analyzed as continuous dependent variables, presented as mean ± SD for normally distributed data or median (interquartile range) for non-normally distributed data. Normality of the data was assessed using a Shapiro-Wilk test. The changes in skin biophysical properties, WSRS and VAS from baseline visit to final follow-up visit were analyzed using a general linear model (GLM) for a repeated measures model. The within-subjects factor was defined as the sampling time point. Sex and age were tested as between-subject effects. Levene and Box M tests assessed the homogeneity of the variance and covariance structure of the dependent variables. The sphericity test of the residual covariance matrix was assessed using Mauchly’s sphericity test. The assumption of the sphericity test is met when *p*-value is more than 0.05. All statistical analysis satisfied the sphericity assumption, and thus the *F*-statistic is valid and appropriate. Results were considered significant if *p* < 0.05 with a 95% confidence interval. Statistical analysis was performed using SPSS 26.0 (IBM Corp., New York, NY, USA) for MacOS.

## 3. Results

### 3.1. Characteristics of Participant

Fifty participants with no background of chronic skin disease, and not undergoing any medication or supplementation targeting skin condition, have been recruited into this study. No dropout during the 3-month prospective follow-ups. Most participants were at the age of 21 to 30 years old and 31 to 40 years old, with the same percentage of 34%, followed by the smallest age group which is more than 40 years old (*n* = 16, 32%). There were more female participants (*n* = 33, 66%) as compared to male participants (*n* = 17, 34%) (Table 1).

### 3.2. Changes in Skin Barrier Function

All the parameters related to skin barrier function showed significant changes (*p* < 0.001) after three-month supplementation of RC, except skin pH (Table 2). Most parameters do not show significant difference when age and sex were tested as between-subject effects. Significant reduction (*p* < 0.001) in TEWL was observed in cheek, neck, and arm, with a reduction of 36.9%, 47.1%, and 39.2% respectively. Consequently, stratum corneum hydration was also significantly improved (*p* < 0.001) towards the end of this study, with 22.8%, 16.3%, and 31.9% improvement at the cheek, neck, and arm area, respectively. Likewise, sebum production was significantly improved (*p* < 0.001) by 115.3%, 183.6%, and 219.1%, at the area of cheek, neck, and arm respectively. No significant changes were observed on skin pH; skin pH was well maintained within the range of 5.7 to 6.1 throughout the study. Results showed that VAS score as self-administrated by participants was significantly improved (*p* < 0.001) from 5.33 ± 1.27 to 7.08 ± 1.31 after RC supplementation, which is equivalent to 32.8% improvement. Age was found to be the factor that influencing the outcomes of TEWL at arm area, and sebum production at cheek and neck area. Statistical outcomes indicated that participants at older age were more responsive towards the effect of RC supplementation.

### 3.3. Changes in Skin Firmness and Elasticity

**Figure 2:**
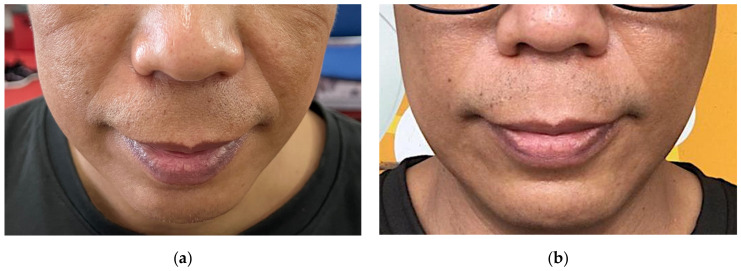
Photographic images of nasolabial folds of a representative patient (**a**) before supplementation; WSRS grade 3, and (**b**) at 3 months after rice ceramide supplementation; WSRS grade 2.

Skin firmness and elasticity of cheek, neck, and arm areas were all significantly improved (*p* < 0.01) after three-month of RC supplementation (Table 3). Viscoelasticity unit (VEU) of cheek area was improved from 0.562 ± 0.134 to 0.662 ± 0.131, equivalent to 17.8% improvement. Similarly, VEU of neck was improved from 0.775 ± 0.065 to 0.829 ± 0.073, equivalent to 7% improvement. VEU of arm was improved by 4.1%, from 0.785 ± 0.042 to 0.817 ± 0.056 at the end of this study. Wrinkle severity as assessed by WSRS also showed a significant reduction (*p* < 0.01) from 1.92 ± 0.89 to 1.60 ± 0.78, which is equivalent to 16.7% change. Photographic images of nasolabial folds before and after RC supplementation are presented in Figure 2. Photographic assessments reported changes from grade 3 WSRS (moderately deep nasolabial fold) before supplementation to grade 2 WSRS (shallow but visible nasolabial fold with a slight indentation) after supplementation.

### 3.4. Changes in Skin Pigmentation

Results showed that both MI and EI of participants were significantly reduced (*p* < 0.001) in all the three areas in the end of this study (Table 4). The changes in MI and EI illustrated a clear time-dependent pattern. The reduction of MI in the area of cheek, neck, and arm were 13.2%, 17.7%, and 15.2%, respectively. Likewise, EI of participants was significantly reduced (*p* < 0.001) by 15.7%, 15.2%, and 12.5% at the area of cheek, neck, and arm respectively.

## 4. Discussion

Skin is the largest organ exposed to external environments and is easily affected by changes in extrinsic factors. The effect of UV radiation on skin health is well documented, along with other factors such as visible light exposure, circadian biology, dietary intake, smoking, and air pollution [21,22,23,24,25]. Recent studies reported that exposure to visible light led to skin hyperpigmentation, even on dark-skinned individuals [26,27]. Similarly, intrinsic factors such as aging, genetic predisposition, hormonal changes, diabetes, or vascular disease contribute to dynamic skin health [28,29]. Plant-based supplementations, commonly known as natural products, have recently gained much attention and popularity due to their safety compared to animal or synthetic products [30,31]. Oral intake of plant-based ceramides was well documented in animal studies and several human studies, though these studies primarily focus on inflammatory response and dermatitis conditions [9,13,32]. This study is the first to report the beneficial effects of RC supplementation on skin health status via comprehensive conduct of clinical assessments.

Our findings revealed that RC supplementation effectively improves the overall skin health of participants. Multiple indicators of skin barrier function have shown significant improvement throughout the study, with some showing evident effect as early as four weeks after RC supplementation. Those parameters included TEWL, stratum corneum hydration, and sebum production. Sebum production showed the most noticeable change after three months of RC supplementation. The improvements were 115.3%, 183.6%, and 219.1% at the cheek, neck, and arm, respectively. Although specific roles of sebum are yet to be fully established, the composition of sebum suggests its possible role in formation of skin barrier, anti-fungal properties, anti-bacterial properties, and moderation of cutaneous inflammation [5,33]. Disruption of sebum compositions has been associated with various cutaneous diseases such as acne vulgaris, atopic dermatitis, psoriasis, and rosacea [34]. The underlying mechanism of sebum production induced by RC remains unclear, but it is believed to be associated with the increase in ceramides production following RC consumption. Ceramides are known to enhance lipid synthesis in sebocyte culture models [35]. Interestingly, sebum production in all three examined areas improved drastically to the normal healthy range, from 70 to 180 for cheek, 55 to 130 for neck, and >6 for arm [36,37]. Older participants in our study showed more significant cheek and neck sebum production changes than those from younger age categories. It is most probably their extremely low sebum secretion rate at baseline that renders them more responsive to the effects of RC supplementation [38].

There are two main skin surface lipids, sebaceous lipids derived from sebaceous gland and epidermal lipids derived from stratum corneum. These skin surface lipids play a crucial role in maintaining healthy skin homeostasis [39]. Our results indicated that RC supplementation exerts a suppressive effect on TEWL, most probably attributed to the improved production of sebum and ceramides. The higher content of ceramides in stratum corneum is the key factor in upregulating epidermal differentiation, thereby limits the transmission of water [40]. Factors attributing to water retention are water supply from the basal layer, sebum production, water loss through evaporation, protein coating of the cells, and presence of lipids in extracellular matrix [41]. These factors concurred with our present findings indicating inversely proportional changes between skin hydration and TEWL. Despite various significant changes in skin parameters of our study participants, their skin pH remained within the healthy range, oscillating between 5.7 and 6.1. Similar to the findings from previous studies, higher skin pH for our participants was recorded in the more hydrated area, which is the cheek, followed by the neck, and lastly the arm [42]. The significant improvements in sebum production and skin hydration, as well as reduction in TEWL suggested that RC supplementation effectively restored the skin barrier function. As reflected by VAS scoring, the skin condition of participants improved by 32.8% towards the end of this study.

Skin condition is often influenced by intrinsic factors such as TEWL, water hydration, sebum production, and dermal elasticity [43]. All these factors are interconnected and highly susceptible to changes among them. Previous studies reported sebum production to be closely associated with elasticity, which is in agreement with findings in this study [44]. An increase in sebum production leads to the augmentation of skin softness and improves skin elasticity. These improvements are likely caused by protective effects of skin surface lipid layer against UVB-induced skin damage, thereby improving skin hydration, and appearance of wrinkle [15]. Ceramides administration was also proven to be effective in downregulating the enzymatic activity of matrix metalloproteinase-1 (MMP-1) and MMP-13, a collagenase family, while simultaneously improving the biosynthesis of procollagen type I [15,45,46]. The suppression of MMPs is likely due to the upregulation of tissue inhibitors for metalloproteinases (TIMPs) in the presence of high ceramides [46]. The improvement in skin firmness and elasticity were further verified using WSRS photogenic assessment, in which 30% of the participants exhibited apparent improvement in wrinkle severity after three months of RC supplementation.

In addition to the role of ceramides in maintaining the healthy skin barrier function, ceramides are also known to be involved in various cellular events as second messengers, such as cell proliferation, cell cycle arrest, senescence, and apoptosis [47]. Hence, it is hypothesized that ceramides may play a role in regulating melanocytes, and evidently, several studies supported the possible role of ceramides in depigmentation [16,48]. Likewise, our findings also revealed that RC supplementation exerts a significant effect in reducing the MI of participants. Although the underlying mechanism of depigmentation is not well defined, but several studies have suggested the potential role of ceramides in regulating tyrosinase activity [49]. Ceramides were reported to have a suppressive effect on tyrosinase gene expression [50]. A study investigating the melanocyte cell model suggested that ceramides can directly inhibit tyrosinase activity and reduce melanocyte growth via delayed activation of the Akt/PKB pathway [48]. Moreover, the potential role of ceramides in circadian rhythms and physiology as signal mediator could also contribute to the alternation of melanocyte function [51,52].

Erythema index (EI) represents the amount of haemoglobin in one particular area. The measurement principle was based on the absorption peak of haemoglobin at 660 nm wavelengths. Skin erythema, commonly known as a flare, is the reddening response of skin upon exposure to external stimuli such as an allergen [53], or UV radiation [51,53]. Our results indicated that RC supplementation effectively reduces participants’ EI. Although ceramides were known to promote inflammatory responses, new evidence suggests otherwise [54,55]. In vitro study demonstrated that ceramides can effectively reduce the expression of inflammatory enzymes and cytokines, predominantly inducible nitric oxide synthase (iNOS), cyclooxygenase-2 (COX-2), and tumor necrosis factor-α (TNF-α) by inhibiting NF-κB activation [54]. On top of that, skin barrier function is known to be the critical factor in regulating inflammatory responses. It is well documented that impaired skin barrier function contributes to the development of sensitive skin, making it more prone to inflammation [56]. It is reported that an impaired barrier function can activate transient receptor potential vanilloid-4 (TRPV4), which triggers inflammation and immune responses [57]. It is noteworthy that defective skin barrier function is also linked to structural changes permitting easy access to allergens, pathogens, and toxic environmental pollutants, subsequently causing systemic inflammation [58]. Therefore, it is conceivable that inflammatory response can be reduced by restoring the skin barrier function, which is in line with our research findings.

## 5. Conclusions

Although the efficacy of topical ceramides application in improving skin barrier function was well documented, scientific evidence supporting clinical efficacy of oral administration were still limited and often focused on rather specific parameters. The present work is the first to study the efficacy of oral RC supplementation in improving skin health via full-scale skin parameters assessments. Our findings concluded that RC supplementation is effective in inducing sebum and ceramides production, leading to improved skin barrier function. Along with that, inflammatory response, wrinkle severity, skin firmness, and elasticity also showed noticeable improvement during this study. Interestingly, our findings also revealed the potential use of RC supplementation for skin whitening effect. No adverse events were reported during this study and skin pH was well maintained in the range of 5.7 to 6.1. Nonetheless, the lack of a placebo group is an important limitation of this study. Randomized controlled trials are recommended for future studies to validate our findings.

## Figures and Tables

**Table 1:** Characteristics of Participant.

Characteristic	Frequency (%)
Sex (*n*/%) Male Female	17 (34.0) 33 (66.0)
Age (years) (*n*/%) 21–30 31–40 >40	17 (34.0) 17 (34.0) 16 (32.0)

**Table 2:** Changes in skin barrier function of participants during study.

Parameters	Baseline	First Follow-Up	Second Follow-Up	Third Follow-Up	^a^*p*-Value	^b^*p*-Value	^c^*p*-Value
TEWL (g/h/m^2^)							
Cheek	21.23 ± 5.01	14.20 ± 6.15	14.10 ± 6.59	13.39 ± 5.06	<0.001 *	0.652	0.185
Neck	18.38 ± 5.13	11.44 ± 2.47	10.50 ± 1.19	9.72 ± 2.23	<0.001 *	0.524	0.396
Arm	14.45 ± 5.51	9.59 ± 3.84	8.32 ± 3.18	8.79 ± 3.22	<0.001 *	0.677	<0.05 *
Skin Hydration (AU)							
Cheek	61.10 ± 14.95	60.65 ± 14.50	66.53 ± 13.94	75.05 ± 14.80	<0.001 *	0.544	0.605
Neck	58.07 ± 16.40	57.78 ± 13.06	60.81 ± 14.42	67.56 ± 15.77	<0.001 *	0.475	0.096
Arm	38.96 ± 12.98	38.17 ± 10.35	45.25 ± 12.62	51.40 ± 13.97	<0.001 *	0.625	0.078
Sebum Production (AU)							
Cheek	35.18 ± 10.33	51.6 ± 20.40	68.34 ± 19.21	75.74 ± 28.48	<0.001 *	0.425	<0.01 *
Neck	23.40 ± 5.69	37.54 ± 12.11	53.34 ± 22.83	66.36 ± 22.36	<0.001 *	0.935	<0.05 *
Arm	1.78 ± 0.45	3.00 ± 0.10	4.60 ± 0.21	5.68 ± 1.04	<0.001 *	0.23	0.316
Skin pH							
Cheek	6.02 ± 0.34	6.09 ± 0.37	6.10 ± 0.44	6.01 ± 0.33	0.475	0.066	0.936
Neck	5.83 ± 0.29	5.86 ± 0.30	5.95 ± 0.38	5.89 ± 0.32	0.226	0.497	0.952
Arm	5.73 ± 0.33	5.77 ± 0.27	5.87 ± 0.35	5.79 ± 0.27	0.112	0.986	0.52
VAS	5.33 ± 1.27	5.81 ± 1.29	6.57 ± 1.40	7.08 ± 1.31	<0.001 *	0.41	0.776

TEWL Transepidermal Water Loss; AU Arbitrary Units; VAS Visual Analogue Scale. Statistically significant *p* values are marked in asterisks (*). ^a^ *p*-value was calculated using general linear model (GLM) for repeated measures model, with sampling time point as within-subjects factor. ^b^ *p*-value was calculated using general linear model (GLM), with sex tested as between-subject effect. ^c^ *p*-value was calculated using general linear model (GLM), with age tested as between-subject effect.

**Table 3:** Changes in skin firmness and elasticity of participants during study.

Parameters	Baseline	First Follow-Up	Second Follow-Up	Third Follow-Up	^a^*p*-Value	^b^*p*-Value	^c^*p*-Value
Skin Firmness and Elasticity (VEU)							
Cheek	0.562 ± 0.134	0.606 ± 0.116	0.610 ± 0.111	0.662 ± 0.131	<0.001 *	0.347	0.851
Neck	0.775 ± 0.065	0.782 ± 0.074	0.759 ± 0.092	0.829 ± 0.073	<0.001 *	0.741	0.938
Arm	0.785 ± 0.042	0.789 ± 0.060	0.795 ± 0.063	0.817 ± 0.056	<0.01 *	0.578	0.994
WSRS	1.92 ± 0.89	1.78 ± 0.88	1.70 ± 0.86	1.60 ± 0.78	<0.01 *	0.499	0.74

VEU Viscoelasticity Unit; WSRS Wrinkle Severity Rating Scale. Statistically significant *p* values are marked in asterisks (*). ^a^ *p*-value was calculated using general linear model (GLM) for repeated measures model, with sampling time point as within-subjects factor. ^b^ *p*-value was calculated using general linear model (GLM), with sex tested as between-subject effect. ^c^ *p*-value was calculated using general linear model (GLM), with age tested as between-subject effect.

**Table 4:** Changes in skin pigmentation of participants during study.

Parameters	Baseline	First Follow-Up	Second Follow-Up	Third Follow-Up	^a^*p*-Value	^b^*p*-Value	^c^*p*-Value
Melanin Index (MI) (AU)							
Cheek	175.72 ± 60.86	171.77 ± 58.26	162.79 ± 60.24	152.58 ± 55.31	<0.001 *	0.325	0.093
Neck	137.50 ± 39.98	128.05 ± 48.19	126.53 ± 51.41	113.20 ± 43.96	<0.001 *	0.727	0.116
Arm	135.27 ± 46.34	131.07 ± 40.99	127.53 ± 39.27	114.74 ± 37.35	<0.001 *	0.163	0.09
Erythema Index (EI) (AU)							
Cheek	329.80 ± 79.83	312.00 ± 87.18	301.46 ± 80.80	278.00 ± 82.49	<0.001 *	0.078	0.25
Neck	255.53 ± 70.95	246.35 ± 66.49	237.78 ± 64.30	216.76 ± 60.47	<0.001 *	0.184	0.096
Arm	196.22 ± 57.68	192.43 ± 52.72	182.94 ± 43.94	171.67 ± 51.69	<0.001 *	0.104	0.836

AU Arbitrary Units. Statistically significant *p* values are marked in asterisks (*). ^a^ *p*-value was calculated using general linear model (GLM) for repeated measures model, with sampling time point as within-subjects factor. ^b^ *p*-value was calculated using general linear model (GLM), with sex tested as between-subject effect. ^c^ *p*-value was calculated using general linear model (GLM), with age tested as between-subject effect.

## Data Availability

The protocols of this study were registered in clinicaltrials.gov (accessed on 1 November 2021) with identifier NCT05101421.
